# Enhanced Lymphatic Uptake of Leflunomide Loaded Nanolipid Carrier via Chylomicron Formation for the Treatment of Rheumatoid Arthritis

**DOI:** 10.15171/apb.2018.030

**Published:** 2018-06-19

**Authors:** Yadhu Krishnan, Shilpa Mukundan, Suresh Akhil, Swati Gupta, Vidya Viswanad

**Affiliations:** Amrita School of Pharmacy, Amrita Institute of Medical Sciences and Research Centre, Amrita Vishwa Vidyapeetham, Kochi – 682041, India.

**Keywords:** Rheumatoid arthritis, Lymphatic uptake, Nano lipid carriers, Leflunomide, Chylomicron

## Abstract

***Purpose:*** The current study aims the lymphatic delivery of leflunomide loaded nanostructured lipid carriers (LNLC) for the treatment of rheumatoid arthritis, mainly focussed to enhance the lymphatic delivery via chylomicron formation, improved bioavailability and reduced systemic toxicity.

***Methods:*** Melt emulsification ultra-sonication method was used to formulate the nanostructured lipid carrier (NLC) containing leflunomide. Four batches were prepared by using various concentration of surfactants (tween 80 and poloxmer 188) and lipid mixtures (stearic acid and oleic acid). All the formulations were studied for various physiochemical properties

***Results:*** The formulation with increased concentration of lipid and surfactants showed highest entrapment efficiency (93.96 ± 0.47%) and better drug release (90.35%) at the end of 48 hrs. In vivo tests were carried out to determine the antiarthritic potential of the formulation in Sprague-dawley rats for a duration of 30d. The effect was evaluated by measuring the reduction in knee thickness. LNLC showed a marked reduction in inflammation compared to standard drug. Intestinal lymphatic uptake studies of LNLC were performed by intraduodenal administration and compared with leflunomide drug solution. The mesenteric lymph node was analysed by HPLC method and the concentration of drug was estimated. It showed that LNLC having highest uptake (40.34μg/ml) when compared with leflunomide drug solution (10.04μg/ml). Radiographic analysis and histopathological studies showed the formation of healthy cartilage after treatment period.

***Conclusion:*** The results suggested that LNLC has the potential to reduce the systemic toxicities associated with conventional therapy along with improved efficacy in the treatment of rheumatoid arthritis.

## Introduction

Rheumatoid arthritis (RA) is an autoimmune disease, which results in disabilities due to progressive inflammation & destruction of joints.^1^ It is characterized by inflammation of synovial joint, production of auto antibodies, bone & cartilage deformities. The cause of RA is unknown, but it is believed that both genetic & environmental factors contribute RA.^2^ Advances in understanding the pathogenesis involved in RA & developing drugs which target them brought a revolution in the treatment of RA. Different classes of drugs are used for the treatment, in which some are used to ease the symptoms & others are used to slow or stop the disease activity. Non-steroidal anti-inflammatory drugs (NSAIDs), disease modifying anti-rheumatic drugs (DMARDs), corticosteroids & biologic agents are the drug classes used for the treatment of RA. DMARDs are a class of anti-rheumatic drugs which not just treats the symptoms associated with the disease but acts on the immune system & modify the disease itself.^3,4^ The treatment of RA with currently available DMARDs is based only on empirical observations.^5^ Methotrexate, leflunomide, sulfasalazine & hydroxychloroquine are the most commonly prescribed DMARDs.^6^ Leflunomide, a pyrimidine synthesis inhibitor, is a leading drug among DMARDs.^7^ Leflunomide acts by modifying the inflammatory processes, particularly in RA.^8^ The primary challenge in the treatment of RA with leflunomide is its systemic side effects including hepatotoxicity, allergic reactions etc. A site specific delivery of the drug can reduce these systemic side effects. Lymphatic system, which plays a great role in the pathogenesis of RA, is considered to be one of the effective delivery sites for the treatment of RA. A site specific delivery into the lymphatic system can be achieved by nano based drug delivery systems unlike conventional delivery systems. Thus the systemic and unwanted side effects can be avoided.^9^

For patient compliance, oral route of drug administration is mostly preferred.^10^ But the oral route is having some limitations due to the physicochemical properties of the drugs including low permeability & solubility, instability & rapid metabolism which results in decreased bioavailability. Most of the newly discovered drugs are having low solubility & high permeability belonging to BCS class-II. The bioavailability of orally administered lipophilic drugs is limited due to these characteristics.^11^ The oral delivery of drug can be improved by drug transport through intestinal lymphatic system. NLCs, SLNs, liposomes & emulsomes are novel drug delivery systems designed to deliver drugs through the lymphatic systems & lipid based drug delivery systems are considered to be the best.^12^

## Materials and Methods

Leflunomide was obtained as a gift sample from aarti pharmaceuticals, mumbai. Stearic acid as solid lipid purchased from nice chemicals, kerala, oleic acid as liquid lipid & tween 80 as surfactant was purchased from loba chemie pvt. Ltd. mumbai. Poloxamer 188 used as surfactant was purchased from research lab fine chem industries, mumbai. Methanol from nice chemicals, kerala. Adult female Sprague dawley rats weighing 200-250 g was obtained from central lab animal facility, AIMS, kochi.

### Formulation of leflunomide loaded NLC

LNLC was prepared by melt emulsification ultrasonication method.^13^ In this method the aqueous & oil phases were prepared separately & then mixed together. Aqueous phase was prepared by dissolving desired amount of surfactants in water under heating at 80°C with continuous stirring. Simultaneously lipid phase was prepared by melting a blend of solid lipid and liquid lipid at 80°C & the drug was dissolved in the melted lipid. Then the aqueous phase was added drop wise into the lipid phase at the same temperature by the aid of agitation at 600 rpm for 10 m & the pre emulsion obtained is further sonicated using a probe sonicator to form NLC. The optimization of the formulation was carried out using different ratios of lipids and surfactants which is mentioned in Table 1. To compare the particle size and zeta potential a formulation of leflunomide loaded SLN was also prepared by the same method without the incorporation of liquid lipid.

**Table 1:** Composition, particle size, zeta potential, polydispersivity index and percentage entrapment efficiency of drug loaded LNLC

**Batch code**	**Drug (mg)**	**Stearic acid (%)**	**Oleic acid (%)**	**Tween 80 (%)**	**Poloxamer188 (%)**	**Particle size (nm)**	**PDI**	**Zeta potential**	**Entrapment efficiency (%)**
**F1**	10	3.5	1.5	0.75	0.75	91.93±0.02	0.381±0.02	-25.3±2.7	93.96 ± 0.47
**F2**	10	3.5	1.5	0.25	0.75	92.54±17.00	0.607±0.12	-17.4±1.4	89.40 ± 0.50
**F3**	10	3	1.5	0.75	0.75	177.33±13.05	0.378±0.01	-21.8±4.0	91.86 ± 0.26
**F4**	10	3	1.5	0.25	0.25	45.66±3.34	0.0.452±0.06	-10.2±1.7	85.98 ± 0.52

### Characterization of leflunomide loaded NLC

#### Particle size and Zeta potential

Particle size and zeta potential of LNLC was determined by differential light scattering (DLS) using a computerized inspection system by zeta sizer.^14^ A particle size ranging 10-100 nm is found to be optimal for lymphatic uptake and retention in lymph node.^15^

#### Scanning electron microscopy and X-Ray diffraction

The morphological evaluation of LNLC was done by using scanning electron microscope (SEM). Information about the surface composition and topography of the samples will be obtained from the images produced by SEM. The sample was diluted & a drop of it was mounted on an aluminium stub with double sided adhesive carbon tape, it was dried & evaluated with SEM. The crystallinity and melting behaviour of the lipid nanoparticles were analyzed by DSC. Leflunomide API and LNLC were subjected to DSC analysis. The analysis was performed at a heating rate of 10°C/min between 30.0-300.0°C. XRD (X-ray diffraction) provide information on unit cell dimension used for phase identification of crystalline material. XRD analysis leflunomide API and lyophilized LNLC powder was performed in the range 2 θ. XRD data confirms the results of DSC analysis.

#### Determination of Entrapment efficiency

The entrapment efficiency was calculated by the indirect method in which the concentration of free drug in the aqueous phase of the dispersion was determined.

$$\text{Entrapment efficiency }\left(\text{\%}\right)\text{= }\frac{\text{Ct-Cf}}{\text{Ct}}\text{×100}$$

Ct – concentration of total amount of the drug added

Cf – concentration of free drug

In this technique, the LNLC was centrifuged at 10000 rpm at 5°C for 1 hr. After centrifugation, the supernatant obtained was diluted with 5ml methanol & the concentration of free drug (leflunomide) in the solution was quantified by UV spectroscopy analysis at 260nm. The study was performed in triplicate & the value was expressed as mean standard deviation.^11^

#### *In vitro* release study

*In vitro* drug release of entrapped drug (leflunomide) in NLCs was studied in phosphate buffer saline (PBS) of pH 7.4 using dialysis membrane. The dialysis membrane was activated by washing with running water for 3-4 h and treated with 0.3% sodium sulphide for 1m at 80°C followed by washing with hot water at 60°C for 2 m and acidified with 0.2% w/v sulphuric acid for a period of 1 m and finally rinse with hot water and stored in distilled water at refrigerated condition. PBS 7.4 was used as the dissolution medium. In order to determine the cumulative drug release, formulation containing 1mg of drug (2.5ml) was placed in an open end dialysis tube suspended in 30ml of phosphate buffer solution pH 7.4 at 37±1°C which was stirred at 50rpm. At predetermined time intervals, the samples were withdrawn and replaced with same amount of fresh medium. The withdrawn samples were analysed spectrophotometrically at 260 nm.^14^ Triplicate measurements were taken and results are expressed as mean standard deviation. The values that are obtained from the *in vitro* drug release study can be quantitatively analysed by using different mathematical formulae. The data obtained from *in vitro* drug release studies were plotted in various kinetic models: - Zero order drug release kinetics, first order release kinetics, Higuchi model and Korsmeyer peppas model.

#### Stability study

The optimized LNLC formulation was stored at room temperature (at 25°C) and refrigerator temperature (4-8°C) for a period of one month. Then the formulation was analysed for particle size (PS), polydispersity index (PDI) and entrapment efficiency (EE).

### Haemocompatibility Analysis

The blood samples (5ml) was collected from healthy donor & immediately mixed with acid citrate dextrose (750μl) to prevent clotting. Then the blood sample was diluted with phosphate buffer saline (pH 7.4) in the ratio of 1:9. To the diluted blood samples different concentrations of nanoparticle formulation was added & incubated for 24 h. After 24 h, the sample was centrifuged at 3500 rpm for 10 min at 4°C. The supernatant was collected & transferred to a micro titre plate & taken absorbance at 540 nm using an Elisa plate reader. 0.1% triton X-100 treated blood sample was used as positive control.^15^

### In vivo anti-inflammatory studies

*In vivo* anti-inflammatory studies were conducted on adult female Sprague dawley rats weighing 200-250 g. Four groups of animals having six each were housed in polypropylene cages, temperature maintained at 23±2°C. Lighting has to be controlled to supply 12 hrs light & 12 hrs dark for each 24 hr period. Chronic Arthritis will be induced by an intra-articular injection of 0.1 ml, of concentration 1 mg/ml complete freund’s adjuant (CFA). Procedures were performed under isoflurane anaesthesia. After the development of arthritis-carrier, drug and formulation (0.1% w/v each) will be administered orally twice a week with an interval of 3 d between the doses for a period of 30 d to group II, III & IV respectively. The knee circumference (mm) of each group was measured from Day 1 - Day 30 using a digital micro meter. Data obtained was used to calculate percentage inflammation at different time points.^16^

#### Lymphatic uptake study by HPLC method

Sprague-dawley rats were selected for the *in vivo* intestinal lymphatic uptake study. The animals were divided into two groups, group-1 administered with standard leflunomide solution and group-2 with optimized leflunomide NLC formulation (LNLC) (F1). The tail vein of each rat was cannulated with a polyethylene tube under isoflurane anaesthesia. A small incision was made in the abdomen, the duodenum is located, and the LNLC formulation and drug solution was injected directly using a 1 mL syringe with a 31-gauge needle. The whole gastrointestinal tract will be then carefully replaced in the abdominal cavity, and the incision closed using clamps and kept moist by covering with gauze pads pre-soaked in normal saline. The rat was warmed using a lamp, and normal saline was infused at a rate of 1.5 mL per h during the experiment to rehydrate the rats. 1 h after administration of the LNLC and leflunomide drug solution, the rats were sacrificed, and isolate mesenteric lymph node. The mesenteric lymph nodes obtained was weighed and homogenized with four volumes of phosphate buffered saline using a homogenizer. After centrifugation, 200 µL aliquots of homogenate transferred to a new 2 mL tube, and add 1.5 mL of methanol.^17^

#### Estimation of liver enzymes (ALT, AST and ALP)

Liver injury can be diagnosed by certain biochemical markers like alanine aminotransferase [ALT], aspartate aminotransferase [AST] and alkaline phosphatase [ALP]. The blood samples for liver function test were given at merit labs, palarivatton, kochi, kerala. Elevations in serum enzyme levels are taken as the relevant indicators of liver toxicity. In this study, the liver toxicity was estimated based on the level of liver enzymes by using liver function test. Macroscopic and in particular histopathological observations and investigation of additional clinical biochemistry parameters allows confirmation of hepatotoxicity.

### Histopathological studies

Histopathological analysis of liver samples of control, standard and test animals were carried out to confirm the ability of leflunomide NLC in reducing the liver toxicity, the systemic toxicity problem associated with pure drug. At the end of the study (after 30 d) the knee joints of standard and test group was subjected to histopathological analysis and compared with that of a normal animal’s knee joint in order to confirm the better effectiveness of LNLC as anti-arthritic drug than leflunomide API.

## Results and Discussion

### Particle size and Zeta potential

Leflunomide loaded NLC was prepared by melt emulsification ultra-sonication method. A particle size of 10-100 nm was observed to be optimal for lymphatic uptake & due to the net negative charge of interstitial matrix, negatively charged lipid based drug delivery systems are reported to be showing higher lymphatic uptake compared to neutral & positively charged surfaces. The retention time of particles in lymph node increases with increase in negative charge.^18^ In this study, F1 NLC formulation is found to be having a better PDI with an average particle size of 91.93 ± 4.70 nm and zeta potential -30 mV. As mentioned in Table 1 particle size is increased with high lipid content and increased surfactant concentration leading to higher viscosity of continuous phase resulting broadness of size distribution.^19^ The prepared SLN were found to have a particle size of 257.14 ± 5.14nm and zeta potential of -26mV.

### Scanning electron microscopy

**Figure 1:**
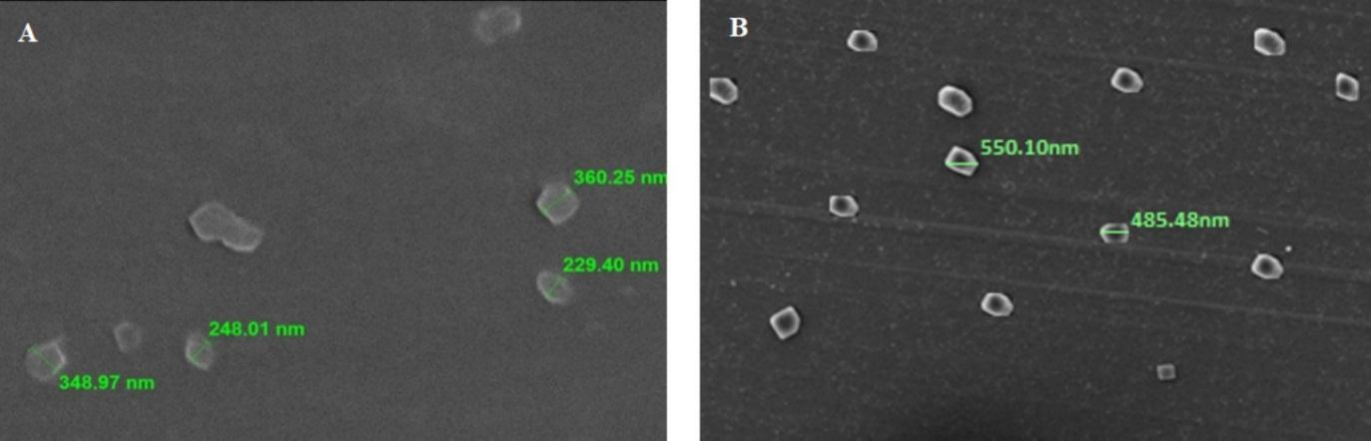
A: SEM image of leflunomide NLC and B: SEM image of leflunomide SLN

The morphological characters of the optimized formulation F1 was evaluated using scanning electron microscopy (SEM) analysis. Figure 1A shows the SEM photograph of LNLC which indicates that the particles were spherical and no drug crystals were visible. NLC aggregation has led to a particle size of 250nm while observed under SEM. Figure 1B shows the SEM photograph of LSLN with a particle size of more than 500nm due to aggregation.

### Differential scanning calorimetry and X-ray diffraction

**Figure 2:**
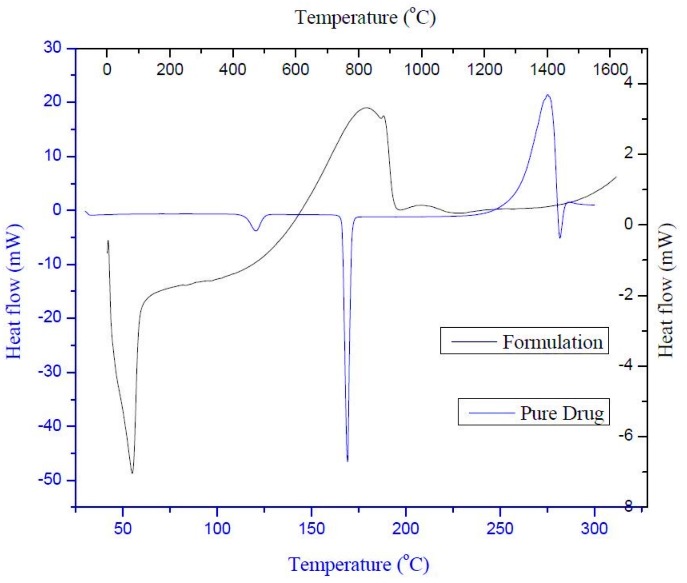
Differential scanning calorimetry of pure drug and drug loaded NLC formulation

The sharp peak at 167.03°C corresponds the melting peak of leflunomide was shown in Figure 2. The absence of endothermic peak within the melting range of leflunomide in the thermogram of LNLC formulation. Figure shows a complete solubilization of leflunomide in lipid matrix or transformation of leflunomide crystal to amorphous form that has been dispersed in the lipid matrix.^20^

**Figure 3:**
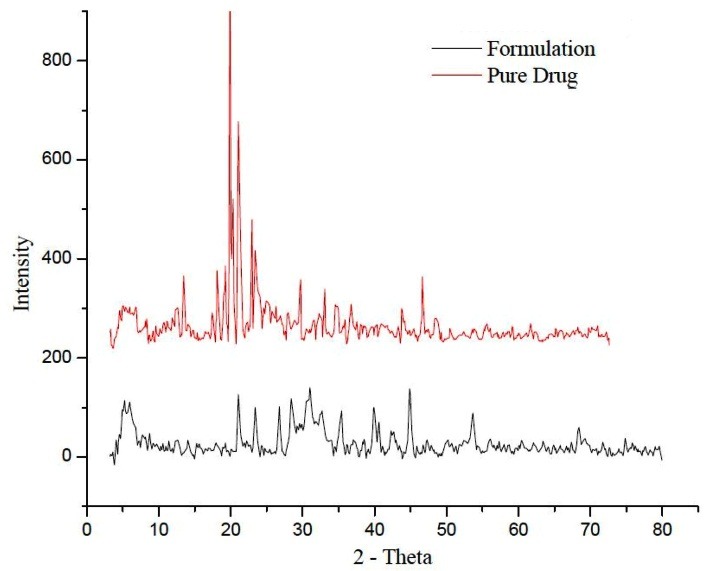
X-ray diffraction analysis of pure drug and drug loaded NLC formulation (F1)

The XRD of leflunomide in Figure 3 exhibits intense lines and characteristic peaks which indicates its crystallinity whereas XRD of lyophilized LNLC of F1 formulation in figure shows declined peaks indicating the possibility of converting crystalline to amorphous form.^21^

### Entrapment efficiency

The percentage entrapment efficiency of the four different formulations (F1, F2, F3 and F4) was determined and mention in Table 1. Formulation F1 is having the highest entrapment efficiency of 93.96 ± 0.47%. Entrapment efficiency of the formulations was found to be increased with increase in concentration of lipids and surfactants, because an increase in lipid content provide more space for incorporation of drugs and also reduces the tendency of drugs to escape into the external phase.^17^ Surfactants increases the solubility of drugs in lipids, therefore entrapment efficiency will be increased with an increased concentration of surfactants.^18^

### In vitro release study

**Figure 4:**
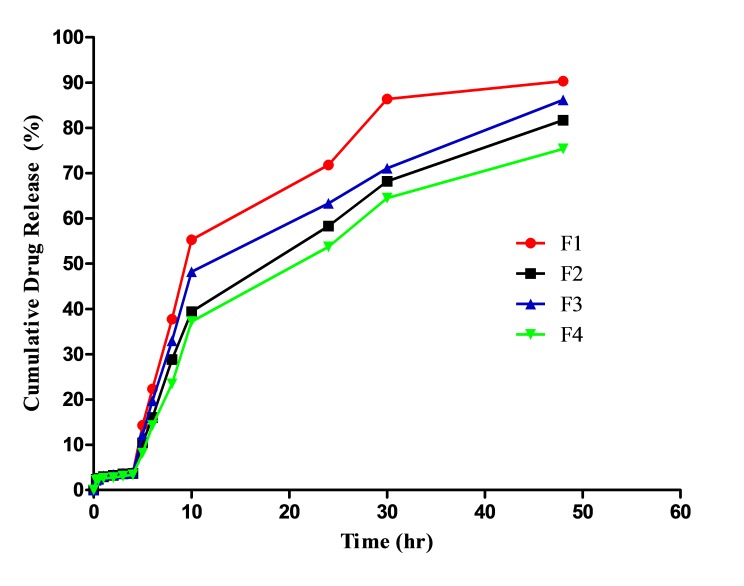
*In vitro* drug release of formulations F1, F2, F3 and F4

The *in vitro* release study of LNLC in phosphate buffer (pH 7.4) was studied for 48 h. The cumulative drug release was calculated and plotted against time. In the present study, the drug was entrapped or solubilized in the lipid matrix and thus lipid enhances absorption and bioavailability of the drug.^22^ Here the highest amount of drug release was observed with formulations having higher lipid concentration. Concentration of surfactants also plays an important role in drug release, since the drug release is found to be increased with an increase in surfactant concentration. The release rate of formulation F2 was found to be decreased with a reduction in concentration of tween-80 showing the significance of surfactants in drug release. Formulation F1 shows a better drug release with 90.35% release rate at 48 hrs. Based on the *in vitro* release studies, formulation F1 was found to be the best formulation for delivering leflunomide into the lymphatic system. Figure 4 shows the *in vitro* drug release of formulations F1, F2, F3 and F4. The data obtained from *in-vitro* drug release study was evaluated by fitting the data into zero order, first order, higuchi model and koresmeyer peppas model based on the R^2^ value the release of the optimised formulation F1 was best fitted in higuchi model and the possible mechanism of drug release might be diffusion of drug from the matrix erosion resulting from degradation of lipids. The n value derived from koresmeyer peppas equation suggest that the mechanism of release follows a diffusion controlled model.

### Stability Study

The purpose of stability study was to provide evidence that the product remains stable for a specified period of time. The particle size (PS), polydispersivity index (PDI) and entrapment efficiency (EE) was measured to ensure that the product characteristics and drug content of the product remain unchanged. At room temperature after seven d, the product shows significant variation in the particle size, PDI and entrapment efficiency whereas at refrigerated temperature after 1 month, product did not show any significant variation. Therefore, from the results, which are mentioned in Table 2, we can say that LNLC formulation is more stable at refrigerated conditions whereas at room temperature it was not found to be stable.^23,24^

**Table 2:** Particle size, PDI and % entrapment of optimized formulation F1 on storage

**Storage condition**	**Particle size (PS)**	**Polydispersity index (PDI)**	**Entrapment efficiency (EE)**
At the time of preparation	86.90 ± 0.327	0.359 ± 0.034	90.62 ± 0.640
Room temperature (RT)	125.80 ± 0.543	0.475 ± 0.294	84.56 ± 0.587
Refrigerated temperature	97.21 ± 0.247	0.379 ± 0.095	87.28 ± 0.810

### Haemocompatibility test 

**Figure 5:**
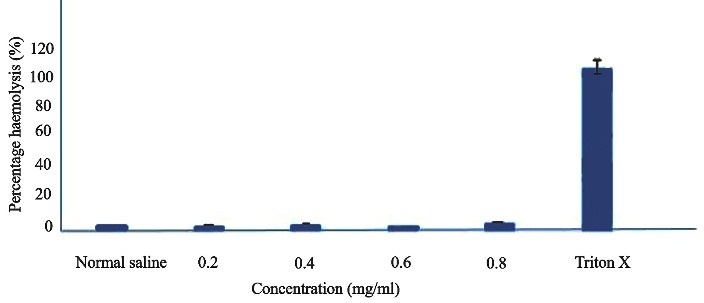
Percentage haemolysis of different concentration of optimized leflunomide loaded nlc in human blood

Haemocompatibility test is carried out to check the compatibility of the drug and formulation with blood, since it came in contact with the blood on *in vivo* administration. The test looks for bursting red blood cells. The degree of haemolysis is a sensitive indicator of the extent of damage to RBC. In the present study, different concentrations of optimized formulation F1 were tested and showed no evidence of haemolysis (<0.5%) after incubation for specified time period.^25,26^ All the concentrations of F1 meet the standard, the data confirmed the optimized formulation F1 provides an acceptable level of haemolysis as shown in Figure 5.

#### In vivo anti- inflammatory study

**Figure 6:**
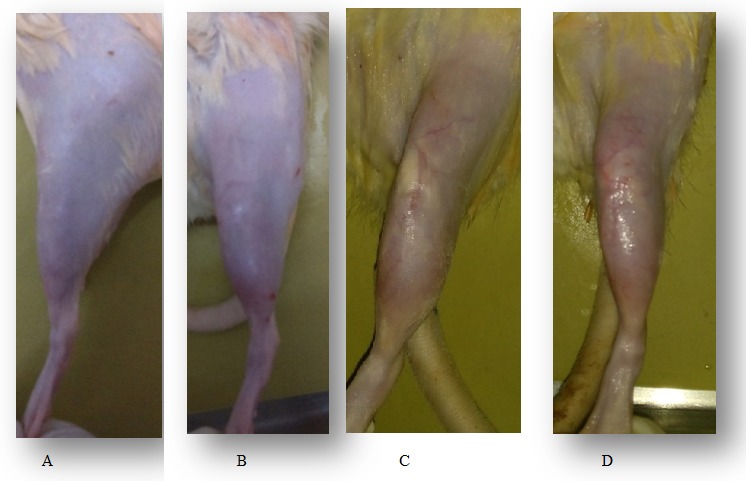
*In vivo* anti inflammatory studies for A) Control (no treatment), B) Carrier (NLC without drug), C) Standard (leflunomide drug solution), D) Test (optimized NLC formulation F1)

**Figure 7:**
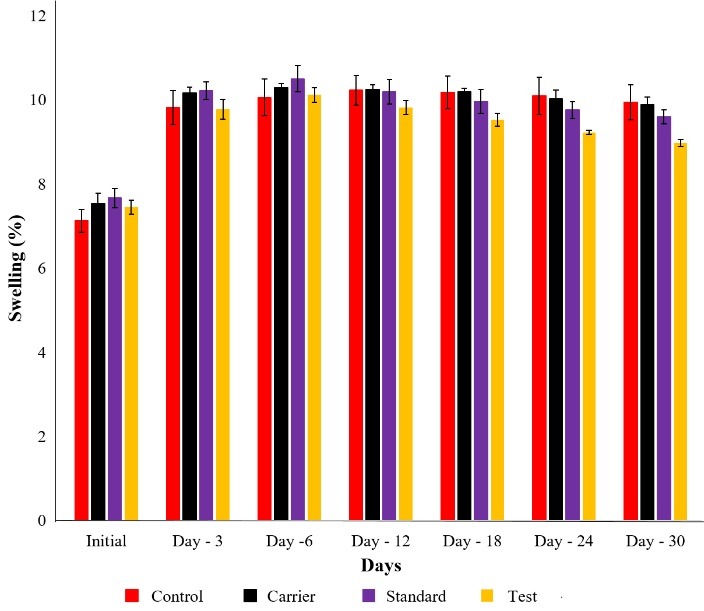
Percentage swelling in CFA induced inflammation

*In vivo* anti-inflammatory study was carried out in Sprague-dawley rats by complete freund’s adjuant (CFA)^27^ induced arthritis for a period of 30 d. The anti-arthritic potential of leflunomide NLC was evaluated by analysing its ability in inhibiting CFA induced knee edema. After the intra-articular injection of 1mg/ml of CFA, inflammation was developed in the left leg, it was then treated by using 0.1 % w/v of leflunomide NLC formulation twice weekly. The effect of the treatment was then evaluated by measuring the reduction in knee thickness which indicates anti-inflammatory action. Knee thickness was measured using digital micrometre was showed in Figure 6. The results of percentage knee edema after 30 d treatment was calculated and results shown in Figure 7. The standard and test group showed a marked reduction in inflammation over 30 d. Whereas the control and carrier group didn’t show a significant reduction in inflammation. Test group showed more reduction in swelling compared to standard group which indicates leflunomide NLC is more effective in anti-arthritic activity than leflunomide drug solution.

### Determination of Lymphatic uptake by HPLC method

**Figure 8:**
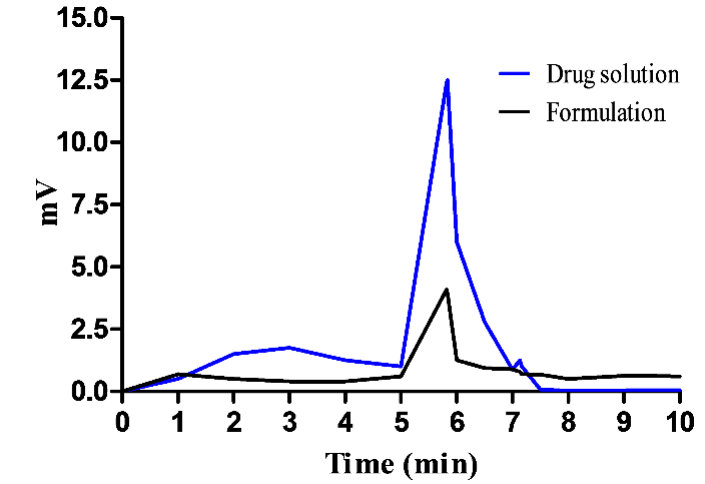
HPLC peak of pure drug solution and drug loaded NLC after lymphatic uptake study

To evaluate the intestinal lymphatic uptake of leflunomide from NLC, the leflunomide content recovered from the mesenteric lymph node at specified time periods after intraduodenal administration of optimized leflunomide NLC formulations to Sprague-dawley rats was measured. The mesenteric lymph node is then analysed by HPLC method and the concentration of drug in the mesenteric lymph node at 1 hr after intraduodenal injection of leflunomide drug solution and leflunomide loaded NLC formulation was found to be 10.04μg/ml and 40.34μg/ml respectively. These results suggest that nano lipid carriers enhance the lymphatic uptake of leflunomide. It is well established that oral NLC facilitate the lymphatic uptake of drugs mainly via chylomicron formation. The results obtained from Figure 8A and 8B showed the significance of NLCs for site specific delivery of drugs into the lymphatic system.

#### Estimation of liver enzymes (ALT, AST and ALP)

Liver function test (LFT) was carried out in control animals and those administered with leflunomide loaded formulation (F1). Since the variation in the normal range of liver enzymes- alkaline phosphatase (ALP), serum glutamic oxalo transaminase (SGOT) or AST and serum glutamic pyruvic transaminase (SGPT) or ALT is an indication of liver toxicity, animals are subjected to LFT. The results as mentioned in Table 3, showed that the ALP and SGPT level found to be elevated in the standard group (leflunomide solution) compared to the test group (leflunomide NLC formulation F1) which indicates that leflunomide drug solution cause damage to the liver whereas in the optimized formulation (F1), the extent of inflammation was reduced and also the effect of leflunomide on liver was found to be in the normal level.

**Table 3:** ALT, AST and ALP level of standard and test group

**Animal group**	**SGOT or AST (IU/L)**	**SGPT or ALT (IU/L)**	**ALP (IU/L)**
**Normal range in rats**	85 - 123	25 - 36	136 - 188
**Standard**	128.9±9.02	40.1±5.8	209.3±10.4
**LNLC**	110.6±12	31.5±5.3	181.1±10.5

### Histopathological studies

**Figure 9:**
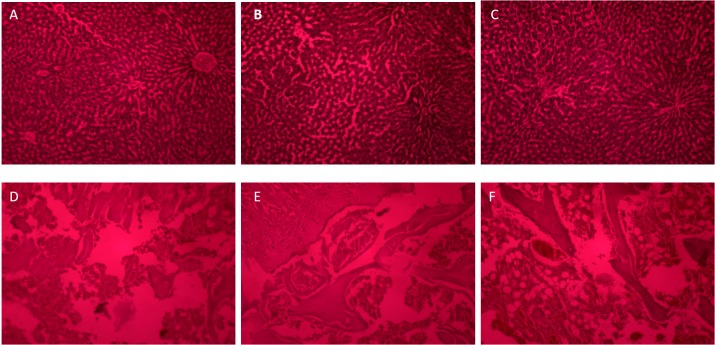
Histopathological analysis of liver samples of Control group (A), Standard group (B) showing liver damage, Test group (C) showing no liver damage. Histopathological analysis of knee joints of Normal group (D), Standard group (E) and Test group (F) seems to be more close to normal when compared with standard.

The histopathogical analysis of liver samples shows no evidence of liver toxicity in test group and the section of standard group shows liver damage since about 30% of hepatocytes show vacuolated cytoplasm, remaining hepatocytes are normal. Small necrotic areas are seen; these areas are replaced by inflammatory cells. Sinusoidal spaces appear congested whereas the section of test group shows normal structure. Portal traids and hepatic veins are normal. Some of the hepatocytes are vacuolated. Sinusoidal spaces and kupffer cells appear normal (Figure 9).

The histopathological study of a normal knee joint of Sprague dawley rat and that of standard and test group was carried out and the results shows the better efficacy of leflunomide NLC in the test group compared to the standard group. In the histopathological section of normal knee joint shows bony tissue with thin bony trabeculae. Osteocytes are normal. Cartilage also appears normal. Marrow is normocellular. Perosteum and surrounding tissue appear normal. Section of standard group shows thin bony trabeculae. Osteocytes are normal. Covering cartilage tissue also appear normal. Peristeum shows fibrosis and there are many collections of lymphocytes, plasma cells and polymorphs and in the test group, section shows bony trabeculae with normal osteocytes. Covering of cartilaginous tissue appear normal. Periosteal tissue shows fatty tissue with dense infiltrate of lymphocytes, plasma cells and polymorphs. The histopathology results of knee joints in Figure 9 shows that the test group section gives similar observations as that of the normal knee joint section compared to standard group.

## Conclusion

Leflunomide is a disease modifying anti-rheumatic drug, which is reported to be equally effective as methotrexate in the treatment of rheumatoid arthritis. Conventional dosage forms of leflunomide are associated with systemic adverse effects and undergo first pass metabolism on oral delivery. In this study, leflunomide loaded nano lipid carrier (LNLC) for site specific delivery into lymphatic system through oral route was prepared, characterized and studied for various *in vitro* and *in vivo* evaluations. The study showed that, the lipids and surfactants used in the preparation has significant impact on particle size and surface charge which further influence the lymphatic uptake of the formulation. *In vitro* drug release studies confirmed the prolonged action and *in vivo* anti-inflammatory study proved the ability of drug loaded formulation to reduce systemic toxicities and improved anti-arthritic activity compared to conventional dosage form. The drug uptake study in mesenteric lymph node provides evidence for the capability of NLC for delivering drugs into intestinal lymphatics through oral route. It can be concluded that leflunomide loaded nano lipid carriers (LNLC) are better candidates for treating rheumatoid arthritis since it reduces the systemic side effects and improve the treatment efficacy compared to conventional dosage form of leflunomide.

In future, the synovial cell line study on SW982 can be done with the optimized formulation F1 to determine the anti-inflammatory effect of the drug. Through the *in vivo* animal study fluorescent imaging can be carried out to confirm the lymphatic uptake of drug loaded nano lipid carrier.

## Ethical Issues

Animal approval was obtained from Central lab animal facility, AIMS, kochi. CPCSEA\Reg No 527\02\A\CPCSEA Dt 21\01\2002, renewal number – 527\PO\ReBi-S\Re-L\02\CPCSEA Dt 30\03\2017 (Ref No: IAEC\2016\1\7).

## Conflict of Interest

The authors declare no conflict of interest. Authors are thankful to Amrita institute of medical sciences and research centre for proving the facilities for doing the work.
